# Facilitating planned home death: A qualitative study on home care nurses' experiences of enablers and barriers

**DOI:** 10.1111/jan.16171

**Published:** 2024-03-21

**Authors:** Anne Kristine Sørstrøm, Mette Spliid Ludvigsen, Ingjerd Gåre Kymre

**Affiliations:** ^1^ Faculty of Nursing and Health Sciences Nord University Bodø Norway; ^2^ Department of Clinical Medicine ‐ Randers Regional Hospital Aarhus University Aarhus Denmark

**Keywords:** end‐of‐life care, home care nurses, home care services, home deaths, palliative care, planned home deaths

## Abstract

**Aim:**

The aim of this study was to explore home care nurses' experience of enablers and barriers for planned home death in municipal health care.

**Design:**

A focused ethnography.

**Methods:**

This qualitative study collected data from 20 semi‐structured interviews of home care nurses and 8.5 h of participant observations. Data was analyzed using thematic analysis.

**Results:**

The findings in our study show that home care nurses consider supportive cultures, a commitment to safety and continuity when facilitating planned home deaths and family rotations to be enablers for planned home deaths. Barriers to planned home deaths involve a lack of palliative experience affecting confidence, shortages of nurses and medical supplies and night shift challenges.

**Conclusion:**

This study underscores the need for supportive organizational cultures, ongoing education and improved communication and staffing policies to enhance the quality of care and the experiences of patients and home care nurses, especially in the context of planned home deaths.

**Impact:**

The study adds knowledge to the evidence base of the practice of facilitating planned home deaths. The findings of the study could offer valuable insights for shaping future policies or devising effective implementation strategies.

**Reporting Method:**

Adherence to the COREQ guidelines for reporting qualitative research was maintained.

**Patient or Public Contribution:**

No patient or public contribution.

**What Does this Article Contribute to the Wider Global Clinical Community?:**

- Identified enablers and barriers provide a new perspective, contributing to a comprehensive understanding of planning home deaths.
- The study emphasizes supportive cultures, safety commitment and family rotations as crucial for planned home deaths, guiding healthcare professionals to adopt best practices and enhance palliative care quality.

## 1 INTRODUCTION

The location of death plays a crucial role in shaping the experiences of both patients and caregivers (Cross & Warraich, 2019). Given the choice, many individuals would prefer to die at home (Burge et al., 2015). However, various sociodemographic factors, including marital status, family support, caregiver preferences, living arrangements and the nature of the disease course, may influence the place of death (Gisquet et al., 2015). Home care nurses (HCNs) play a pivotal role in providing care for these patients, often facing unique and sensitive situations within the privacy of people's homes (Adamson & Cruickshank, 2013). While it is not a novel concept for nurses to deliver care in private residences, the increasing desire for home deaths means that more HCNs will encounter unfamiliar care scenarios (Mørk et al., 2018).

Understanding the experiences of HCNs in facilitating planned home deaths is essential for the development of policies and services aimed at ensuring high‐quality palliative care in the home. We advocate for further exploration of HCNs experience of enablers and barriers when a patient wishes to die at home.

## 2 BACKGROUND

Palliative care aims to alleviate the suffering of both patients and their families during the final stages of life and the palliative care continuum can span from 12 months before death, to bereavement support for next of kin after the time of death (Slåtten et al., 2010). This also includes end‐of‐life care, during the final phase (Slåtten et al., 2010). Palliative care involves a thorough evaluation and management of the physical, psychosocial and spiritual symptoms that patients may encounter (Hagan et al., 2018). As a patient nears the end of life, their symptom burden may increase, necessitating more assertive palliative interventions. As the measures to ensure comfort become more intense, so does the assistance extended to the family of the dying patient (Rome et al., 2011). After the patient's passing, the primary emphasis of palliative care shifts towards providing support to the bereaved family (Rome et al., 2011). In recent years, palliative care has gained ground in research and recommendations voice a greater emphasis on the place of care before death. The broadening of the scope of palliative care, from a predominant focus on cancer and end‐of‐life care to encompassing diverse diagnoses and early palliative interventions, is anticipated to increase the number of palliative patients (Helsedirektoratet, 2017).

Utilization of homecare services is on the rise in Norway, and this trend is projected to continue (Mørk et al., 2018). Patient preference plays a significant role in determining the place of death (Burge et al., 2015; Cai et al., 2020). Consequently, some authors argue that ensuring death occurs in the preferred place is a more fitting indicator of quality than the percentage of home deaths (Billingham & Billingham, 2013). Many individuals who have a short time left to live wish to die at home. However, the needs of the individual may change over the course of their illness. Therefore, facilitating more time at home can be just as important as facilitating death at home (Helse og omsorgsdepartementet, 2020).

Earlier studies found that patients' likelihood of passing away at home is primarily influenced by factors such as diminished functional condition, personal preferences, utilization and intensity of home care, living circumstances and the presence of extended family support (Gomes & Higginson, 2006). More recent studies report factors associated with home death over the hospital as patient preference, family choice, use of home palliative care and involvement with home care nursing in the final 3 months of life (Gomes et al., 2015). However, a global needs assessment survey from 2019 found obstacles that hinder the provision of home care, encompassing personnel scarcities, inadequate funding and policy frameworks, limited availability of palliative or hospice services and diminished community awareness regarding available services (Brant et al., 2019). Nurses in the survey pinpointed time constraints, funding shortages and inadequate coverage as the foremost challenges for education in palliative care (Brant et al., 2019). The occurrence of planned home deaths is linked to the involvement of home care nursing (Kjellstadli et al., 2018). In Norway, the job description for HCNs includes the responsibility of delivering high‐quality palliative care to patients within the comfort of their homes during the final stages of life (Helsedirektoratet, 2019). A prerequisite for dying at home is the desire of both the patient and their relatives. Relatives who take on the task of being present are seen as a crucial factor. Accessible and competent nurses must be available to meet the patient's needs. Continuous access to healthcare around the clock is fundamental, as seriously ill and dying individuals have complex needs requiring comprehensive services. Assistance must be readily available on short notice, as changes can occur rapidly. Home care services face challenges in providing palliative care, including professional competence, sufficient time and practical arrangements (Fjørtoft, 2016).

Continuity in care in home care services is of great importance, referring to the role HCNs play in mapping and monitoring patients over time (Hudson et al., 2019; Luker et al., 2000). Understanding the experiences and perspectives of HCNs regarding the facilitation of planned home deaths is important for several reasons. Firstly, HCNs play a pivotal role in providing palliative care at home and their insights into the barriers and enablers are invaluable for improving the quality of care provided. Secondly, by identifying these factors, necessary improvements in services can be implemented, addressing specific challenges faced by HCNs in supporting planned home deaths. Finally, this knowledge is essential for ensuring that the preferences of individuals who desire home deaths are respected and facilitated appropriately, contributing to a more patient‐centred approach in palliative care.

While studies have examined the role of HCNs in providing palliative care at home, we have been unable to trace studies that specifically delve into the identification of enablers and barriers perceived by HCNs for planned home deaths. Hence, the aim of this study was to explore the experiences of HCNs regarding the barriers and enablers they encounter when facilitating planned home deaths.

## 3 METHODS

### 3.1 Design

This study is a subset of a broader qualitative PhD project focused on examining how HCNs facilitate planned home deaths. Previously, we released findings detailing how HCNs contemplate and carry out planned home deaths, including a more in‐depth exploration of the methodological assessments employed (Sørstrøm et al., 2023). This qualitative study is based on data from the above‐mentioned focused ethnographic fieldwork. Reigada, Sapeta and Centeno's review from 2019 emphasizes the importance of ethnographic studies in palliative care. The primary methods employed in most studies include interviews, participant observation and field notes. Ethnographic research analyzes cultural aspects, group relationships, interactions, participant meanings and perceptions, communication processes and language use in specific natural contexts. They conclude that ethnographic methods are valuable for analyzing phenomena, particularly when the context is well‐defined (Reigada et al., 2019). The ethnographic aim of revealing the common elements within a culture, encompassing values, beliefs, knowledge, skills and actions, aligns with the aims of this study (Wall, 2015). Using a diverse range of research methods, especially observational techniques, allows for a comprehensive exploration of nurses' clinical practices. Roper and Shapira's qualitative framework for focused ethnography design (2000) proved suitable for our study. Fieldwork included short‐term and targeted data collection sessions, incorporating participant observations and individual interviews with HCNs. This research design facilitated an examination of perceived enablers and barriers in HCNs' efforts to facilitate planned home deaths. Focused ethnography was chosen for its ability to provide in‐depth insights into HCNs' experiences related to specific phenomenon and their interactions within their environment (Cruz & Higginbottom, 2013). The first author has formal training in palliative care and clinical experience in palliative care settings. However, she is not associated with the home care service where the study took place. All three authors are registered nurses with substantial experience in qualitative research.

### 3.2 Study setting and recruitment

The study was conducted in two municipalities in northern Norway with 50,000 and 80,000 inhabitants respectively. Based on principles of public welfare, the Norwegian healthcare system delivers free and universal healthcare to residents, with municipalities mandated to provide home nursing services, including integrated palliative care (Sommerbakk et al., 2016). The participants in this study were HCNs from urban and rural areas in Northern Norway. We employed purposive sampling to enlist HCNs from home care services, aiming to secure individuals with specialized knowledge and extensive experience in facilitating planned home deaths. Eligible for inclusion were HCNs who had some experience in facilitating planned home death. Potential participants were selected and invited by the district manager after consultation with the first author.

### 3.3 Data collection

Data collection occurred from March 2019 to March 2020, employing an ethnographic approach to understand HCNs' thoughts and actions in their natural setting. We utilized individual semi‐structured interviews and participant observations to gain insights.

#### 3.3.1 Participant observations

For participant observation, the first author observed HCNs facilitating planned home deaths. AKS had beforehand familiarized herself with Spradley's (1980, p. 78) observation guide for field notes and used these as a checklist for what to observe and note. This included space: the physical place or places, actors: the individuals involved, activity: a series of related actions that people perform, object: the physical things present, action: individual actions that people perform, event: a series of related activities that people engage in, time: the sequencing that occurs over time, goal: what people are trying to achieve, feelings: the emotions felt and expressed (Spradley, 1980). Observation sessions included HCNs' preparations, travelling time and time spent during visits. Due to the sensitive nature of the situations, no notes were taken in patients' homes, but reflective notes and quotes were recorded immediately after visits. Note‐taking was integral throughout data collection and analysis, capturing initial impressions and preconceptions and aiding an audit trail.

#### 3.3.2 Interviews

A key informant participated in a pilot test of the interview guide to assess its content and ensure comprehension. Semi‐structured interviews with a set agenda were employed, allowing flexibility to explore participants' ideas. Interviews were conducted at suitable locations within HCNs' workplaces. Drawing on observations, we developed questions aligning with the study's aim, focusing on enablers and barriers when facilitating planned home deaths. The interview guide (File S1 English Interviewguide) included open key questions such as ‘*What do you consider an enabler for planned home deaths*?’ and ‘*What do you think are barriers for planned home deaths?’*. Probing and follow‐up questions were employed for exploration and clarification. All interviews were audio‐recorded, transcribed verbatim and fine‐tuned based on emerging issues from observations. Despite being given the option, participants chose not to read the transcribed material.

### 3.4 Data analysis

The data consisted of transcribed interviews and field notes from observations. The NVivo 12 software program (QSR International Pty Ltd, 2018) was utilized to assist in sorting and organizing the dataset. The research team opted for a theoretical thematic analysis because it aligned with the research aim for this study. This type of analysis is motivated by the researcher's theoretical or analytical interest in the subject, making it explicitly analyst‐driven (Braun & Clarke, 2006). We wanted to provide a more detailed analysis of specific aspects of the data, i.e. enablers and barriers of planned home deaths, focusing on depth rather than aiming for a comprehensive description. This focused approach facilitated a targeted examination of the data, adhering to the principles outlined by Braun and Clarke (2006). Initially, interviews and observational data underwent separate analyses before being collectively examined. AKS began analyzes during the data collection. Familiarity with the data was achieved through multiple readings of interview transcripts. Initial notes and key points were recorded and codes were generated by systematically coding interesting features, such as text segments of respective enablers and barriers, i.e., ‘You can never achieve 100 per cent safety because stuff can happen, but you have to have a plan and there must be…It is important… that we have enough staff’ was preliminarily coded as C5 ‘Not enough nurses’, which was later allocated under the final theme ‘Shortage of nurses and medical supplies’. Codes were crafted concisely, yet providing ample details to independently convey the shared essence among constituent data, as recommended (Braun & Clarke, 2012; Byrne, 2022). Relevant data for each code was collated and recurring patterns and topics were identified. Overarching themes were created, with related codes grouped into preliminary themes. Coherence and relevance were assessed, with themes refined or combined as needed. Each theme was clearly defined and named to capture essential aspects of the interview data.

In examining observational data, AKS familiarized herself with the data by thoroughly reading the transcribed field notes and making notes of initial thoughts and impressions. Initial codes were generated by identifying and labelling interesting features, patterns, or themes within the data and then coded both explicit and implicit aspects of the data. Themes were searched for by collecting codes that were related to each other and grouping them into potential themes. At this point codes that did not fit in the prospective themes were put in a miscellaneous theme, saving this for later to see if it could become a theme itself or if we would discard them (Braun & Clarke, 2012; Byrne, 2022). We reviewed and defined the identified themes, ensuring they accurately represented the data. Observational themes underwent review and refinement, considering complementarity or contrast with interview themes and adjustments were documented.

Interview and observational themes were synthesized, with a comparison of similarities and differences. Areas of convergence or dissonance were identified and we articulated the implications and contributions of the thematic analysis. Recognizing the iterative nature of thematic analysis, flexibility was necessary to capture the richness of the combined interview and observational data. To ensure a robust and credible study we regularly revisited and refined the analysis. The co‐authors assessed the reliability of AKS's coding and confirmed the validity of the identified themes through analytical discussions. Findings and quotes were translated from Norwegian to English while preserving participants' expressions. Verbatim quotes were employed to illustrate themes and this study emphasizes the importance of constructing an analysis that persuasively presents the argument, avoiding the risk of ‘*anecdotalism*’ in qualitative research (Braun & Clarke, 2006).

### 3.5 Ethical considerations

We received approval for the study from the management of the home care services in each municipality. Prior to interviews and observations conducted in patients' homes, HCNs provided written informed consent. Additionally, to ensure patient privacy and compliance, all patients were pre‐informed and asked for their consent to have a researcher accompany HCNs into their homes. Each patient or their next of kin (NOK) signed an agreement granting us permission to observe the nursing activities within their residences. The data underwent anonymization through the removal of names, locations and the alteration of specific details. Interview transcripts and audiotapes were securely stored in locked files. The study adheres to the principles of the Declaration of Helsinki (World Medical Association, 2013). The study adhered to the guidelines established by the Norwegian Agency for Shared Services in Education and Research, formerly known as the Norwegian Centre for Research Data (NSD) (77/356). An exemption from the duty of confidentiality was obtained from the Regional Committee for Medical and Health Research Ethics in Northern Norway (REC) (2019/605). No further applications were necessary according to REC.

### 3.6 Rigour and reflexivity

To ensure rigour and trustworthiness throughout the study, we adopted a reflective approach, from the development of research questions to data collection and analyses, maintaining the richness of the data. To enhance transparency, we established an audit trail, incorporating a reflective diary, ethical considerations, a methodological journal and data analysis chronologies (Holloway & Wheeler, 2010). This approach was complemented by method triangulation, bracketing, member checking and the inclusion of thick descriptions. Member checking involved soliciting feedback from HCNs during and after interviews and field visits, ensuring that observations, notes and assessments aligned with HCNs' beliefs and intentions. This method aimed to minimize decontextualization and misinterpretation of HCNs' descriptions in the analysis process, demonstrating the veracity of the findings. Additionally, adherence to the COREQ guidelines for reporting qualitative research was maintained (Tong et al., 2007; File S3).

## 4 RESULTS

In total, 20 HCNs were interviewed, 17 of whom were women. Their ages ranged from 25 to 63 years (mean 42) and they had 1–24 years of experience (mean 12). There were variations in the degree of experience in facilitating planned home deaths, from two cases to more than ten cases. Thirteen HCNs had specializations, including palliative care (*n* = 2), cancer care (*n* = 8) and others (*n* = 3). Interviews averaged 50 min. The dataset comprises a larger portion of interviews than field notes, primarily attributable to limited occurrences of planned home deaths available for observation during the data collection period. In total, there were seven different observation sessions: four with the HCN, NOK and the patient present, two with only the HCN and NOK and one with just the HCN. These observation sessions were visits to the patients' homes, bereavement conversations and one session in a car with an HCN obtaining supplies for a patient and making phone calls related to the planned home death. Table 1 shows the participants and length of observations.

**TABLE 1:** Overview of performed observations in the study presented as observation number, participants being observed and time of observations in minutes.

Observation number	Participants at observation	Time of observations in minutes
Observation 1	HCN, patient and NOK	60
Observation 2	HCN, patient and NOK	90
Observation 3	HCN, patient and NOK	120
Observation 4	HCN, patient and NOK	60
Observation 5	HCN	30
Observation 6	HCN and NOK	60
Observation 7	HCN and NOK	90
Total		510

*Note*: File S4 Table of observations.

Abbreviations: HCN, home care nurses; NOK, next of kin.

**FIGURE 1:**
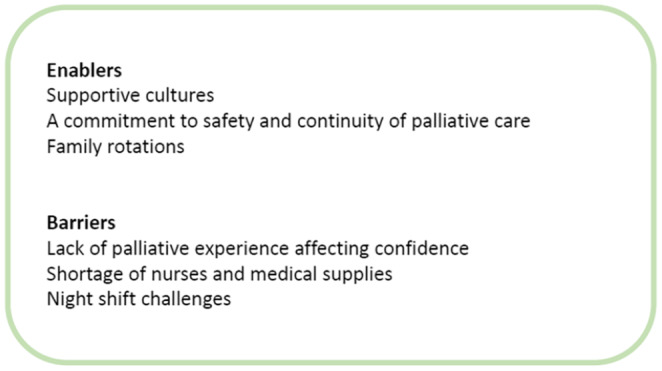
Identified enablers and barriers (File S2: Figure 1 Enablers and Barriers).

We identified various enablers and barriers influencing how HCNs facilitated planned home deaths. Enablers include supportive cultures, a commitment to safety and continuity of care when facilitating planned home deaths and family rotations. Barriers to planned home deaths involve a lack of palliative experience affecting confidence, shortages of nurses and medical supplies and night shift challenges (Figure 1). The analysis identified enablers and barriers affecting HCNs in facilitating planned home deaths at both organizational and individual levels. Organizational factors encompassed issues such as nurse availability, fragmented healthcare services and limited resources. Individual factors included the emotions of HCNs, their confidence levels and perceptions of the NOK' role during the process. Notably, some themes exhibited overlap, as anticipated, given the interconnected nature of these issues.

### 4.1 Supportive cultures

Variations in the incidence of planned home deaths and the culture among HCNs were evident across different home care areas. Notably, in a rural setting, HCNs reported a higher frequency of planned home deaths, reflecting a proactive approach. The language employed by these rural HCNs conveyed a strong sense of collective responsibility and dedication to facilitating planned home deaths. This supportive culture fostered a commitment that went beyond the norm, motivating nurses to go the extra mile, such as taking double shifts to ensure patients could pass away in the comfort of their homes.

Furthermore, distinctions were observed among home care areas regarding the prevalence and characterization of planned home deaths. Specifically, in a rural home care region, HCNs reported a significantly greater number of planned home deaths compared to their urban counterparts. The language used by rural HCNs reflected a higher degree of unity, employing terms like ‘we’ and ‘us’ more frequently than nurses in urban areas. Their descriptions portrayed the active promotion and advocacy of this service within their community, as evidenced by quotes emphasizing the commitment to facilitating planned home deaths.

I feel like we go above and beyond here. We all have that mentality, wanting to make it (planned home deaths) happen. We know it is just for a time, people rolling up their sleeves and taking double shifts when it is going on. Because you know that, you know, we made it happen, we got this done and then things slow down afterwards. (HCN4)

Other HCNs from the home service area that were perceived as proactive by HCNs themselves stated:

Here at this unit, we are… my opinion is that we do a good job in facilitating home deaths. We have leaders who are very understanding and aid us in making it happen. We can do pretty much anything. We have the competence. (HCN18)

We have a strong sense of cooperation and open discussions among professionals here. Our leader is phenomenal; she encourages us to work together and learn from one another, which makes it less intimidating. I have never felt intimidated when going to someone who wants to die at home. Having the support and backup from my colleagues makes a significant difference. We excel at supporting each other and that's why we function so well as a team. (HCN16)

Fieldnote 2:

When following the HCN to a patient and NOK, she brings the work phone but leaves it in the hallway in the patient's house. I asked her after we leave, what if a colleague wanted to get in contact with her while she was making the house call? She says she had told her colleagues where she was and that they don't call when she is with a dying patient. Someone else takes any inquiries or handles situations regarding other patients, if necessary.

These HCNs belonged to the same unit, which had a significantly higher number of planned home deaths compared to the others included in the study. This suggests the presence of distinct subcultures within the home care services across municipalities, with certain areas demonstrating a positive and proactive approach to facilitating planned home deaths.

### 4.2 Commitment to safety and continuity when facilitating planned home deaths

While some HCNs were enthusiastic about gaining more experience, others expressed concerns about the feasibility and safety of planned home deaths, particularly in the context of limited resources and competencies. One HCN commented that planned home deaths were not desirable or recommended, due to the combination of the complexity of the patients' health and the number of nurses available. She had participated in only two planned home deaths over the course of 8 years and she emphasized the concept of accountability in her arguments, as shown in the following excerpt:

AKS: How many home deaths do you have here?

HCN19: You know what, we have very, very few.

AKS: What does that mean? Like one case per year?

HCN19: No, even less. Even less. Because…And I think the biggest challenge is that the patients are in such poor conditions and that exceeds our capacity as professionals. It's a question of professional accountability…we can't do it.

The majority of HCNs emphasized that lack of formal competencies and experience served as barriers to planned home deaths. Additionally, there was a unanimous agreement that the scarcity of planned home deaths hindered their ability to gain valuable experience. Some of the more experienced HCNs noted that the intervals between each case of planned home deaths were increasing over time, suggesting that there were more frequent cases in the past, going back around 10–15 years.

HCNs described how the distinctive aspect of home care lies in the fact that nurses work independently in environments that are not specifically tailored to their tasks. They explained how they upon entering a patient's home, face uncertainty and must adapt to the challenges of the situation as they arise. This sets home care apart from other healthcare settings.

They can see that we don't have the tools to do a good job. And that is important for patients and next of kin. The numerous times I arrived and thought; I don't know anything, I have to tread lightly not to appear as a total idiot, really and thus make them feel unsafe (HCN12)

Despite infrequent involvement in planned home deaths, the HCN maintained a seasoned and calm demeanour on field visits:

HCN is calm, collected, caring and talks quiet and respectfully with the patient and the NOK. A peaceful atmosphere. She inserts a catheter so efficiently and quietly, almost without anyone noticing. She tells the NOK to call if she has any questions or notices any changes in the patient. I ask her if she does this a lot (planned home death) because she comes across as so experienced and calm. She says she has participated in one planned home death previously and it has been a while. (Fieldnote 6)

HCNs reported that it can be challenging to estimate how much longer patients will live. According to HCNs, patients nowadays receive numerous medications, including fluids and glucocorticoids, while in the hospital. When these medications are discontinued and the patients are sent home, their health tends to decline rapidly.

Patients may seem stable and then suddenly, their condition deteriorates rapidly after ending treatments… I recall one individual who had been receiving medical treatment for an extended period. When he returned home from the hospital, we saw the end was near, but we envisioned a few weeks. He had a lot of pain while brushing his teeth due to fungal infections. After an evening shift, I went by the store to buy a small toothbrush for children that would perhaps be less painful to use. Unfortunately, he passed away the following morning. (HCN7)

### 4.3 Family rotations

In this study, ten HCNs (_N1‐N4_, _N6_, _N9‐N11_, _N13_, _N15_) named NOK as the most important enabler for making a planned home death happen. They emphasized the importance of having sufficient NOK who possess the ability, motivation and understanding of the extensive effort required. According to HCNs, a planned home death necessitates a *family rotation*, where there are enough family members to support the patient and each other and take on the responsibility for as long as needed.

Typically, patients who wish to die at home have a network of next of kin. It would be highly challenging, perhaps even impossible, if they did not have such support. (HCN3)

One HCN (N8) mentioned that planned home death is a feasible option, but its success depends on the ability of NOK to cope. The support of family members is crucial, as they are the ones primarily responsible for the care, guided and assisted by HCNs:

AKS: Is planned home death a feasible option?

HCN8: Yes, but only if NOK can cope, because it is they that do it, in a way, with guidance and assistance from us, but I can't imagine we could do it for someone living alone.

There was consensus among HCNs that a planned home death is a viable option when there is a strong and committed network of NOK who can provide support. The involvement of family members is seen as essential for the success of this approach.

I would have to say that the most important thing to have when facilitating planned home deaths is NOK and several of them and that they are willing to stay and ‘work’ nights. And enough nurses and competent staff. (HCN7)

### 4.4 Lack of palliative experience affecting confidence

HCNs mentioned that their lack of experience in palliative care and home deaths was attributed to infrequent occurrences of planned home deaths and long periods between each case, especially in home care areas near hospitals. This lack of exposure can affect their confidence in managing complex palliative and end‐of‐life care situations. HCNs emphasized the challenges they faced during their limited experiences but also acknowledged the educational and professional rewards they gained from these encounters.

I know many of us think it would be nice to get more training in planned home deaths, because it happens so rarely that you never get the hang of it. It should happen more often, but the organization hinders it, I think. (HCN10)

On average, the HCNs reported encountering only one planned home death per year and in some cases, even less. The following excerpt provides an illustration of one HCN's most recent experience with a planned home death:

It was so long ago but I remember it being complicated, with many different needs. Wounds and pumps and stuff. So it took some time. And I experienced it as quite scary in many ways. But also incredibly educational and so rewarding professionally knowing that you endured those situations (HCN13)

### 4.5 Shortage of nurses and medical supplies

In this study, all HCNs emphasized how the insufficient number of nurses in the home care services is a barrier. They expressed a strong need for more qualified and knowledgeable staff, as this shortage hindered their ability to provide quality and continuity of care. Several HCNs mentioned being understaffed as a major stressor, compounded by an extensive workload due to an increase in patients under their care, as well as more patients with critical and complex needs. This stressor was exacerbated when there were planned home deaths to facilitate.

We need more hands. We need safe and confident hands that know what they are doing and want to do it. It can't be that home death is scary and that they don't want to do this. So, increasing the (palliative) knowledge, the basal knowledge, in the entire home care service is crucial. (HCN8)

Three of the HCNs cited specific cases where the nurse shortage directly resulted in patients not being able to die at home, despite their expressed desire to do so. They explained that it was emotionally challenging to have to say ‘no’ to patients who wanted to die at home due to staff shortages:

It has happened relatively often I would say that we have had to be upfront and say, ‘We wanted to make it (planned home death) happen, but there aren't nurses on this weekend, so we have to admit you to the short‐term wards’ and it is a horrible feeling (HCN7)

The HCNs extensively discussed the frustrations of balancing the care of patients on their worklists with being flexible and available to meet the individual needs of dying patients. Fulfilling these demands within a designated timeframe was reported as challenging:

It's the communicating with every other instance like doctors, palliative team, the aid centre and having it all add up, because even though someone is dying at home does not mean that everything stops. We have 300 patients. That house of cards, you know. It is challenging. We try to be forthcoming and accessible, but we're stretched pretty thin already.(HCN11)

While accompanying an HCN to obtain medical supplies for a patient, she revealed that they did not have abundant storage of necessities. As a result, they would occasionally negotiate with hospitals or institutions to obtain i.v. fluids and take more than they needed ‘for a rainy day.’ Additionally, she mentioned that it was customary to use coat hangers as i.v. racks. Despite the limited resources, she shrugged her shoulders while sighing and saying, ‘we make ends meet with what we've got.’ *(Field note 4/HCN4)*. Another HCN explained what essential factors were when facilitating a planned home death:

That we have the necessary supplies. Like a bed and medication. Anything they need. Something as simple as a slide sheet. (HCN9)

### 4.6 Night shift challenges

HCNs explained how their perception of the service was that it was fragmented. The fragmentation of services was due to the physical separation of night shift nurses from day and evening shifts. This separation leads to limited communication and information sharing between different units or shifts. HCNs expressed that they often didn't know if there would be a nurse available for night shifts, which raised concerns about the continuity and quality of care provided. HCNs expressed that the separation of night shift nurses could be challenging for patients and their NOK. When patients or NOK need to interact with healthcare professionals during the night shift, they may encounter unfamiliar faces, as there is no physical handover or direct communication.

It is challenging for the patients and next of kin that the night shift is separate because by the time they get involved…if they are to visit, they don't know them. And we don't have a physical handover, so it is sometimes over the phone, but still…That's challenging. (HCN9)

HCNs described feelings of insecurity about the service due to the challenges associated with fragmentation. They mention difficulties in transitions, such as not knowing whether there is a HCN on duty during the night, which could create uncertainty and impact the continuity of care.

The scope of it. It is precarious. Because…there are so many patients, long distances and a lot of colleagues you've never met, just talked to over the phone. And you get insecure, is there a nurse on tonight or not? Those transitions…are difficult. (HCN16)

Because there was no oral shift handover of information between evening and night shifts, healthcare nurses started to doubt the quality of their services. Consequently, this became a hindrance to recommending planned home deaths as an option. As one HCN put it:

Several times, I have thought ‘I should have offered these patients the alternative of dying at home’, but I couldn't make myself do it because we don't have enough people. Because we have staff during the day and evening shifts. But still, the night shift is a different department. (HCN15)

HCNs have highlighted the shortage of staff during night shifts as a flagged concern. When there are no nurses available during planned home deaths at night, patients are forced to resort to emergency rooms or rely on on‐call nurses from local nursing homes. As a result, some HCNs voluntarily made themselves available to be on‐call during nights without getting paid or clearing this with a manager. These observations were noted during the fieldwork:

When we left the patient's house in the evening, I asked her (HCN9) if there was a nurse on during the following night. She said she did not know, but if there was not, she would write her name up as an available nurse to call if any questions or problems arose. I asked her if she had the authority to do that (the time was around 7.30 pm, with no leaders to approve this) and she said she had done the same many times before. But she had never checked to see if she had been given hours for it or been paid for the awake hours. (Fieldnote case 3)

HCNs convey a willingness to be extra flexible and take on night shifts, even if it goes beyond their usual working hours. This highlights a sense of dedication to the patients' needs and a willingness to go above and beyond to ensure that care is provided, as seen in the following excerpt:

Where it is possible, I really think we should offer it, try to make it happen. If that requires us to be extra flexible and take on a night shift…even though I work days and evenings, that would be ok. (HCN7)

## 5 DISCUSSION

The aim of this study was to explore the experiences of HCNs regarding the barriers and enablers they encounter when facilitating planned home deaths. The analysis revealed various enablers, including supportive cultures, a commitment to safety and continuity of palliative care and family rotations. Conversely, barriers encompassed challenges such as a lack of palliative experience affecting confidence, shortages of nurses and medical supplies and difficulties associated with night shifts.

Supportive cultures refer to a strong collective responsibility and commitment to facilitating planned home deaths among HCNs. Ganann, Weeres, Lam, Chung & Valaitis reported similar findings – how the intricacy of patient care added to the challenge of finishing demanding tasks within the designated visit duration, leads nurses to put in extra, uncompensated hours to deliver vital care (Ganann et al., 2019). In this study, empathetic leadership and professional cooperation emerged as key factors when facilitating planned home deaths in proactive home care areas. Supportive leaders who understood the complexity of the process contributed to success. Furthermore, HCNs emphasized positive teamwork and nurturing a supportive culture, reflecting a commitment to collaboration. This indicates distinct subcultures within home care services, with some areas actively promoting planned home deaths while others may not prioritize this service. This cultural variation significantly impacts end‐of‐life care options. It underscores the importance of leadership, teamwork and a cooperative environment in achieving successful planned home deaths within home care services. In previous research, HCNs sought consistent counselling due to the demanding nature of working with palliative care patients, which can be physically and mentally taxing (Danielsen et al., 2018). However, while spontaneous sharing of experiences occurred when time allowed, HCNs emphasized the need for leaders to assume a more prominent role in coordinating regular and systematic mentoring and counselling sessions (Danielsen et al., 2018).

The majority of HCNs in our study expressed a genuine eagerness to facilitate more planned home deaths in their home care areas. They saw this as an opportunity for learning and improving their skills in palliative care at home. This demonstrates a commitment to providing the best possible care to patients in their homes. However, HCN19 had a distinct perspective, expressing reservations about the desirability and feasibility of planned home deaths due to nurse shortages and perceived competence gaps. This emphasis on ‘*professional accountability*’ suggests concerns about compromising patient safety and care quality in the current circumstances. The majority of HCNs also recognized the lack of formal competencies and experience, in addition to nursing shortages, as barriers to facilitating planned home deaths. Other studies have reported a lack of trained providers in the community as a barrier to adequate access to palliative care (Lalani & Cai, 2022) and as a direct cause of reduced relational continuity (Nysæter et al., 2022). Access to and availability of staff resources significantly impact the optimization of home care nursing practices and are linked to HCN retention (Ganann et al., 2019). Inadequate learning opportunities, including time and financial constraints, hindered HCNs' professional development. Heavy workloads further limited their learning. HCNs have identified knowledge and skill gaps in many areas like palliative care and caring for clients with increased acuity (Ganann et al., 2019). Resolving these issues may require continual education, training and broader discussions about the role of home care in palliative and end‐of‐life care, including resource allocation.

A key finding in our study is the concept of family rotations, which implies shared responsibility among family members, helping prevent caregiver burnout and ensuring consistent fulfilment of the patient's needs. Studies have found that informal caregiving plays an important role at patients' end of life (Becqué et al., 2019, 2021; Bjørnelv et al., 2020). In our study, a majority of the HCNs cited family rotations and sufficient NOK willing to take on the day‐to‐day responsibility for the dying patient as being an important enabler for planned home deaths and the absence of this support makes delivering the desired level of care challenging. The number of days spent at home is recognized as a quality measure—a patient‐centred goal and outcome—during the end‐of‐life period (Groff et al., 2016). According to the viewpoint of NOK, the adverse consequences of their role tend to outweigh the positive aspects, impacting their physical and psychological well‐being, employment status and family relationships (Bauer & Sousa‐Poza, 2015). Nevertheless, research suggests that the duration of informal caregiving is correlated with an increase in negative effects on NOK (Bauer & Sousa‐Poza, 2015).

Palliative care training and education are highlighted as major facilitators for palliative care in community settings (Lalani & Cai, 2022). In this study, experienced HCNs noted that the intervals between cases of planned home deaths were increasing over time. This suggests a decrease in the frequency of planned home deaths, which may be attributed to various factors, including changes in healthcare practices and patient preferences. This can imply a vicious circle – they don't have enough experience because the frequency is low, which in turn means they don't gain the necessary experience. However, because they lack experience, it might affect their ability to offer patients the option to die at home and thus becomes a barrier to facilitate this practice. In our study, we found indicators for more planned deaths in the rural areas. A reason for this may be longer travel distances for relatives, making HCNs more reluctant to admit patients into urban hospitals. Similar findings have been found in previous research (Kaasalainen et al., 2011). Another explanation for this can be that when questioned about their confidence in performing various activities related to palliative care, rural nurses indicated significantly higher self‐efficacy compared to their urban counterparts (Kaasalainen et al., 2011). In continuation of this, HCNs emphasized the importance of increasing knowledge and competency in‐home care services. This reflects the need for ongoing education and training to ensure that healthcare providers are equipped to handle the complexities of home care, especially in palliative and end‐of‐life situations. Proficient staff emerged as a key facilitator in a meta‐ethnography in 2018 (Wahid et al., 2018). The accuracy of staff predictions regarding a patient's survival time is crucial for accessing specialist palliative care services and resources. More experienced and knowledgeable staff can identify at‐risk patients, offer precise prognoses and facilitate timely access to essential resources near the end of life. In success stories where patients fulfilled their desire to die at home, bereaved relatives lauded the staff as ‘exceptional, competent and considerate’ (Wahid et al., 2018).

The shortage of nurses and medical supplies adds to the complexity and challenges of providing home care services, especially in the context of palliative care and planned home deaths (Collier et al., 2019). All HCNs in this study emphasized the significant shortage of qualified nurses in home care services. The consequences are particularly acute when dealing with patients with complex needs or palliative care at home and patients not being able to fulfil their desire to die at home. The shortage of nurses and the need to turn down patients who wish to die at home can have a profound emotional impact on HCNs. Literature supports our findings, reporting on HCNs experiencing burnout related to heavy workloads and emotional demands of the work of home care services and how this is exacerbated by challenges in recruitment (Ganann et al., 2019). Findings demonstrate the need for additional resources, staffing, training and support to ensure the delivery of high‐quality care in the home care setting. HCNs discussed the difficulty of balancing the care of their regular patient workload with the need to be flexible and available to meet the unique needs of dying patients. This balance can be challenging, given the volume of patients they are responsible for and the need for coordination with various healthcare teams. Previous studies have reported a negative correlation between time available for nursing and good care routines (Aiken et al., 2002; Slåtten et al., 2010). HCNs in this study noted that patients often receive multiple medications and treatments in hospitals and when they are transitioned home, their health can deteriorate rapidly. This notion is corroborated by existing literature, which highlights that patients being discharged too late is viewed as a significant obstacle (Wahid et al., 2018). Furthermore, the clinical instability of patients, where the period between the cessation of active treatment and the need for a home transfer is too brief, hinders effective decision‐making and results in insufficient preparation for patient discharge (Wahid et al., 2018). On the contrary, studies have shown how prognostic challenges for patients emerged as a notable obstacle to home‐based end‐of‐life care. Patients frequently received discharges with projected life expectancies of only a few days or weeks, but these estimates often proved significantly inaccurate, as many patients surpassed the initially predicted survival duration (Wahid et al., 2018). One study underscored a primary factor contributing to the issue of deaths occurring in the hospital and some patients passing away during transit home – the clinical instability of patients. This instability results in a brief window from the cessation of active treatment to the need for effective decision‐making and home transfer, causing insufficient time for adequately preparing the patient's discharge (O'Brien & Jack, 2010).

Resource limitations in terms of medical supplies and equipment were reported as a barrier in this study. Insufficient accessibility to equipment, services and medications has previously been identified as a barrier to the delivery of optimal palliative care, especially in rural areas (Kaasalainen et al., 2011). The phenomena of HCNs ‘stocking up’ on supplies, such as medications and storing them in the boots of their cars, anticipating the need for additional supplies to handle weekends or unexpected increases in client demands have been described in previous literature (Kaasalainen et al., 2014).

Night shift challenges were notably highlighted in our study, with the fragmentation of services identified as a major barrier. This was attributed to the physical separation of night shift nurses from their counterparts on day and evening shifts. Earlier research has emphasized the significance of availability and relational continuity in palliative home care (Hudson et al., 2019) and how patients in this context express a desire to interact with the same healthcare personnel (Nysæter et al., 2022). Delivering continuity of care is a challenging task, often posing difficulties in measurement. However, it holds immense importance for patients (Hudson et al., 2019). The establishment of personalized care plans is grounded in relational continuity, emphasizing the essential requirement for effective informational and managerial continuity both between and within services (Hudson et al., 2019). The fragmentation and lack of physical handover not only affect healthcare providers but also have an impact on patients and their families. Patients may not be familiar with night shift nurses, which can create challenges in terms of building trust and maintaining continuity of care. A scoping review by Cai and Lalani (2022) described fragmented palliative care systems and attributed this to disparities in healthcare resource availability and care models without well‐established principles of care planning, coordination and transition (Cai & Lalani, 2022). HCNs in our study described feeling insecure about whether there would be a nurse available during night shifts. As a result, the HCN exhibits availability and flexibility, demonstrating dedication that includes voluntarily taking on‐call duty beyond scheduled hours. However, the absence of formal compensation arrangements may have adverse long‐term consequences, such as burnout, operational challenges and a negative impact on work‐life balance. Excessive flexibility and going above and beyond may be a vulnerability, potentially masking the need for additional staff or resources and contributing to nurse exhaustion in the long run (Ervik et al., 2020). While important for planned home deaths, this extreme flexibility could imply non‐sustainable coping mechanisms.

### 5.1 Implications for policy and practice

This research holds notable implications for healthcare delivery models advocating for home‐based care, especially regarding the extensive dependence on NOK for planned home deaths. Acknowledging family challenges, improving communication and involving them in palliative care decisions are necessary.

More research is needed, particularly focusing on promoting supportive cultures in all areas and perhaps especially on how to foster supportive cultures when personnel and medical equipment are scarce. Additionally, alternative solutions to challenges during night shifts should be explored, considering how these can be addressed without the individual HCN’ self‐initiative. System‐level changes, including scheduling and staffing policies, are needed for quality palliative care in the home. A comprehensive approach involving organizational, educational and systemic interventions is necessary to address challenges in providing palliative care at home and thus have implications for planned home deaths.

### 5.2 Limitations

Several limitations warrant consideration. The fieldwork, conducted during daytime and evening hours, excludes insights into planned home deaths during the nights. Additionally, the restricted time in the field, attributed to a low incidence of planned home deaths during data collection, imposes limitations on the study's depth. The context specificity to the Norwegian healthcare system raises concerns about the direct applicability of findings to settings with different structures and resources. Although organizational culture differences are acknowledged, their exploration is not exhaustive. In summary, while the study provides valuable insights into HCNs' perceptions of enablers and barriers in facilitating planned home deaths, limitations in generalizability, palliative care scope and depth of analysis on organizational cultures and workforce challenges should be acknowledged.

To enhance the comprehensiveness of future research, it is recommended to expand the scope of fieldwork to include nighttime observations of planned home deaths. Increased data collection time could mitigate limitations imposed by a low incidence during the study period. Moreover, comparative studies across diverse healthcare systems would contribute to understanding universal and context‐specific challenges.

## 6 CONCLUSIONS

Home care nursing requires clinical proficiency, interpersonal aptitude, adaptability and critical thinking. Navigating diverse patient populations, dynamic home environments, ethical challenges and limited resources, HCNs strive to deliver compassionate, high‐quality palliative care when facilitating planned home death. This study highlights the importance of fostering supportive organizational cultures, continual education and enhanced communication and staffing policies to improve the quality of palliative care and enhance the experiences of patients and healthcare professionals in home care services, particularly during the facilitation of planned home deaths.

## AUTHOR CONTRIBUTIONS

A.K.S, M.S.L and I.G.K: Made substantial contributions to conception and design, or acquisition of data, or analysis and interpretation of data. A.K.S, M.S.L and I.G.K: Involved in drafting the manuscript or revising it critically for important intellectual content; A.K.S, M.S.L and I.G.K: Given final approval of the version to be published. Each author should have participated sufficiently in the work to take public responsibility for appropriate portions of the content; A.K.S, M.S.L and I.G.K: Agreed to be accountable for all aspects of the work in ensuring that questions related to the accuracy or integrity of any part of the work are appropriately investigated and resolved.

## FUNDING INFORMATION

This research received no specific grant from any funding agency in the public, commercial or not‐for‐profit sectors.

## CONFLICT OF INTEREST STATEMENT

The authors declare that there are no financial, personal or other conflicts that could potentially bias or influence the research or its findings.

### PEER REVIEW

The peer review history for this article is available at https://www.webofscience.com/api/gateway/wos/peer‐review/10.1111/jan.16171.

## Supporting information


File S1.

File S2.

File S3.

File S4.

## Data Availability

The data that support the findings of this study are available on request from the corresponding author. The data are not publicly available due to privacy or ethical restrictions.
