# Supplementary material for: Facilitating planned home death: A qualitative study on home care nurses' experiences of enablers and barriers
Source: J Adv Nurs. 2024 Mar 21;81(1):340–52. doi: 10.1111/jan.16171 (PMC11638527; doi:10.1111/jan.16171)
Supplement: Supplementary file 1 — File S1. [file JAN-81-340-s003.docx]

Interview guide for home care nurses

Introduction:

- Information about the project, repeat information about consent and the right to withdraw from the study at any given time
- Topic for the interview. Ask for elaborate answers and rich descriptions
- Feel free to ask questions during the interview if anything is unclear
- “With your permission, this interview will be recorded. The audiofiles will be deleted when the project is completed”

Background:

- Age
- Sex
- Education
- How many years of experience as a nurse and experience as a home care nurse

Questions:

- What are your experiences with planned home deaths?
  - - (How many times, duration, any stories?)
- Can you tell me about a time where it turned out to be a good experience? What made it a good experience?
- Can you describe how you facilitate a planned home death?
- Who do you talk to? Who is involved in the planning?
- What do you consider an enabler for planned home deaths?
- What challenges do you face when facilitating planned home deaths? What do you think are barriers for planned home deaths?
- Do you feel safe and confident meeting patients wanting to die at home?
  - - What will it take for you to feel safe and confident?
- Who do you have to cooperate with when planning a home death?
  - - Does that cooperation work?
- Have you experienced a patient wanting to die at home, where it did not turn out that way?
- Do you feel safe and confident when facilitating a planned home death?
- What to you consider most important when facilitating a planned home death?
- Does any of the home care nurses work more with planned home deaths that others? If so, why?

Sum up:

- Follow-up questions?
- Clarify any ambiguities?
- “Did I understand you correctly when you said that….”
- “Do you have anything more to add?
- How did you experience this conversation?
