# Supplementary material for: Facilitating planned home death: A qualitative study on home care nurses' experiences of enablers and barriers
Source: J Adv Nurs. 2024 Mar 21;81(1):340–52. doi: 10.1111/jan.16171 (PMC11638527; doi:10.1111/jan.16171)
Supplement: Supplementary file 2 — File S2. [file JAN-81-340-s004.pdf]

Enablers
Supportive cultures
A commitment to safety and continuity of palliative care
Family rotations

Barriers
Lack of palliative experience affecting confidence
Shortage of nurses and medical supplies
Night shift challenges
