# Supplementary material for: Facilitating planned home death: A qualitative study on home care nurses' experiences of enablers and barriers
Source: J Adv Nurs. 2024 Mar 21;81(1):340–52. doi: 10.1111/jan.16171 (PMC11638527; doi:10.1111/jan.16171)
Supplement: Supplementary file 4 — File S4. [file JAN-81-340-s002.docx]

**Table 1: Overview of performed observations in the study presented as observation number, participants being observed and time of observations in minutes.**

| **Observation number** | **Participants at observation** | **Time of observations in minutes** |
| --- | --- | --- |
| Observation 1 | HCN, patient and NOK | 60 |
| Observation 2 | HCN, patient and NOK | 90 |
| Observation 3 | HCN, patient and NOK | 120 |
| Observation 4 | HCN, patient and NOK | 60 |
| Observation 5 | HCN | 30 |
| Observation 6 | HCN and NOK | 60 |
| Observation 7 | HCN and NOK | 90 |
| Total |  | **510** |

HCN= Home care nurses, NOK= Next of kin
